# Tuned Oxidation Potential of Continuous Defective Photocatalyst for Selective Biomass Conversion

**DOI:** 10.1002/advs.202523412

**Published:** 2026-03-24

**Authors:** Longfei Hong, Huiyan Zhang, Liangdong Hu, Xiang Gao, Lianhua Xu, Qingyu Liu, Rui Xiao, Sheng Chu

**Affiliations:** ^1^ Key Laboratory of Energy Thermal Conversion and Control of Ministry of Education School of Energy and Environment Southeast University Nanjing China

**Keywords:** biomass valorization, defect energy levels, selective oxidation, wavelength‐controlled photocatalysis

## Abstract

Photocatalytic biomass conversion offers an energy‐efficient, low‐carbon route for the sustainable production of fine chemicals. However, selectivity control of biomass valorization is challenging due to the presence of multiple functional groups and competing reaction pathways. Herein, we report a wavelength‐controlled photocatalytic strategy with adaptive oxidative capability for regulating biomass conversion selectivity. Using black TiO_2_ with continuous defect energy levels as a model photocatalyst, the wavelength‐dependent excitation of defect states confers adaptive oxidative behavior, enabling selective conversion of xylose into high‐value xylonic acid under visible light, avoiding over‐oxidation into low‐value C1 products under UV irradiation. The high selectivity is attributed to the tunable photooxidation capability of defect states, where the oxidation potential of defect states excited by visible light is appropriate to suppress the production of non‐selective highly oxidative hydroxyl radicals. This work reveals the correlation between the defect energy‐level distribution and the selective biomass oxidation behavior, offering a self‐tuning approach for directional biomass conversion through catalyst design. The strategy is further validated across a range of biomass‐derived substrates, highlighting its broad applicability in selective chemical synthesis using adaptive photocatalysis.

## Graphical abstract

This work develops a wavelength‐controlled photocatalytic strategy that utilizes defect‐state excitation to modulate oxidative capability for biomass selective conversion. By tuning the photooxidation potential to match substrate reactivity, the system suppresses overoxidation and enables the selective formation of value‐added products, providing a self‐tuning approach for adaptive and efficient solar‐driven biomass valorization.

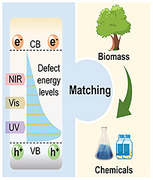

## 1 Introduction

**FIGURE 1:**
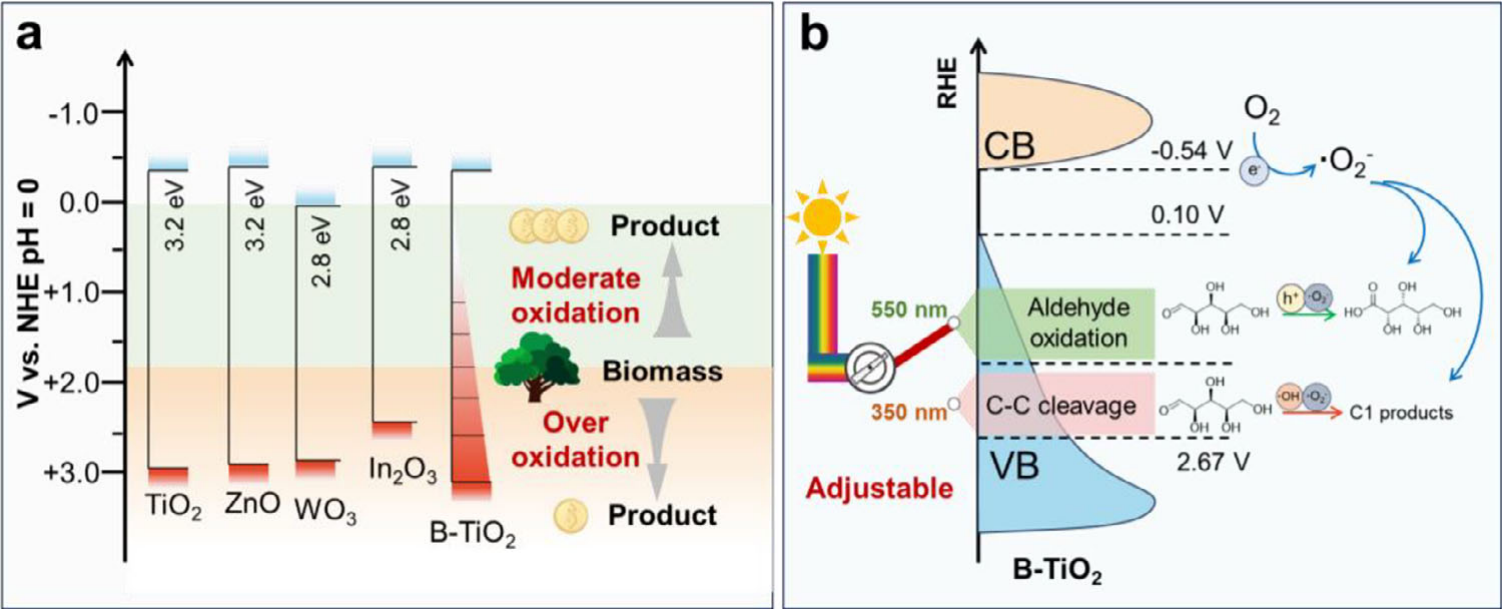
Adaptive defective catalysts for selective biomass conversion. (a). Schematic illustration of the matching between biomass oxidation potentials and photocatalysts with different band structures. (b). Schematic diagram of xylose conversion over B‐TiO_2_ under different wavelengths.

Biomass, as a renewable and carbon‐neutral resource containing diverse functional groups, provides a sustainable feedstock for high‐value chemical production via catalytic conversion [1, 2, 3, 4, 5]. Among them, xylose is a key hemicellulosic sugar whose selective oxidation to value‐added chemicals such as xylonic acid provides a green route to platform compounds and highlights the potential of efficient biomass valorization [6, 7, 8, 9]. However, achieving selective conversion of biomass‐derived intermediates remains challenging due to their complex molecular structures and competing reaction pathways [10, 11, 12, 13, 14]. Conventional thermocatalysis often suffers from poor selectivity control under harsh conditions, leading to undesirable over‐oxidation by‐products. Photocatalysis offers a promising alternative for biomass valorization under mild conditions by harnessing solar energy to generate photoexcited charge carriers for selective conversion [15, 16, 17, 18, 19, 20]. Current commonly used photocatalysts, such as TiO_2,_ possess high oxidation potentials, which tend to cause over‐oxidation of reactive biomass and lead to the formation of low‐value products (e.g., CO_2_) [21, 22, 23]. A fixed band structure corresponds to an unchanging oxidation capacity, thereby limiting its adaptability to different biomass substrates and hindering practical applications in biomass conversion (Figure 1a).

In this work, we reported the use of a defective photocatalyst to obtain wavelength‐dependent adaptive oxidative capability, providing a promising route for controlling product selectivity in biomass valorization. Compared to pristine TiO_2_, black TiO_2_ (B‐TiO_2_), obtained via hydrogenation, introduces defect energy levels that serve as activation centers, enabling self‐adjusting oxidative capability. These defect states not only extend the light absorption range but also open up additional pathways for biomass conversion, making black TiO_2_ a representative model for investigating the relationship between biomass oxidation potentials and photocatalyst band structures [24, 25, 26]. As shown in Figure 1b, under UV light irradiation (350 nm), B‐TiO_2_ tends to over‐oxidize xylose into low‐value C1 products (formic acid, CO, and CO_2_) through C─C bond cleavage. In contrast, under visible light (550 nm), it selectively converts xylose into xylonic acid with a high selectivity of 90.1% and a conversion rate of 2.70 mmol g^−^^1^ h^−^^1^, which are the highest reported for photocatalytic xylose conversion. The defect energy levels in B‐TiO_2_ enable both adaptive oxidation and extended light absorption, supporting wavelength‐dependent control of reaction pathways. The adaptive photochemistry is applicable to various biomass‐derived substrates that are sensitive to oxidation potential. This work establishes a design principle for adaptive photocatalysts by correlating defect engineering, light absorption, and selective biomass conversion, providing a valuable reference for the rational design of smart photocatalytic systems for selective biomass transformation.

## 2 Results and Discussion

### 2.1 Photocatalytic Performance

Xylose is the second most abundant sugar in nature and, with its rich oxygen‐containing functional groups, serves as an ideal feedstock for photocatalytic production of high‐value chemicals [7, 27, 28]. Therefore, we selected xylose as the model substrate to conduct photocatalytic conversion in a fixed‐bed reactor using defect energy level‐rich B‐TiO_2_ as the photocatalyst (Figure S1).

**FIGURE 2:**
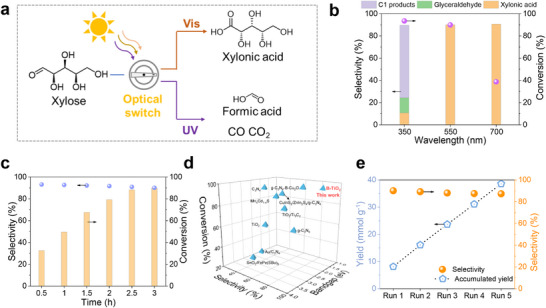
Performance of adaptive photocatalytic systems. (a). Schematic illustration of the optical switch‐controlled biomass conversion pathways. (b). Photocatalytic performance of B‐TiO_2_ for xylose conversion under different light wavelengths. (c). The photocatalytic xylose conversion of B‐TiO_2_ as a function of time. (d). Comparison of B‐TiO_2_’s photocatalytic performance with other catalysts in biomass‐derived saccharides conversion. (e). Photocatalytic stability testing (drying the photocatalyst at 80°C after each run, light intensity: 100 mW/cm^2^, 550 nm).

Firstly, control experiments showed that no xylose conversion occurs in the absence of a catalyst and light. The optimal KOH concentration was subsequently determined to be 0.05 m. (Figure S2). An interesting finding during the photocatalytic reactions is that xylose follows distinct reaction pathways under different light irradiation conditions. As shown in Figure 2a, xylose is converted into low‐value C1 products (formic acid, CO, and CO_2_) with a selectivity of 65.2% under UV light. In contrast, under visible light, xylose is primarily oxidized to xylonic acid (90.1% selectivity). In Figure 2b, the xylose molecules undergo C─C bond cleavage under 350 nm light irradiation, primarily producing C1 products such as formic acid, CO, and CO_2_. The conversion rate of xylose is 93.5%, with a total selectivity of 65.2% toward C1 products, together with the detection of minor by‐products including glyceraldehyde, lactic acid, and oxalic acid. With increasing wavelength, C─C bond cleavage becomes less pronounced, leading to a continuous decline in C1 product formation. Under 550 nm light irradiation, the defect energy levels in B‐TiO_2_ predominantly oxidize xylose to xylonic acid, maintaining a selectivity of over 90% with a similar conversion rate during a three‐hour reaction (Figure 2b,c). Control experiments further confirmed that the sample hydrogenated for 6 h exhibits the optimal balance between activity and selectivity (Figures S3 and S4). Under near‐infrared light (λ = 700 nm), xylose conversion dropped to 38.7% due to limited driving force (Figure 2b). Pure TiO_2_ showed similar performance to B‐TiO_2_ (92.9% conversion, 73.1% C1 selectivity) at 350 nm, but illustrated no activity under 550 or 700 nm light due to its wide bandgap (∼3.2 eV) (Figure S5) [29, 30, 31, 32].

The B‐TiO_2_ catalyst used in this study was treated with hydrogen, resulting in the formation of defect energy levels. As shown in Figure 2d, compared with previously reported systems, the B‐TiO_2_ catalyst not only enables efficient visible‐light‐driven transformation through defect‐state excitation under mild conditions, but also demonstrates outstanding performance in the selective conversion of biomass‐derived monosaccharides. In particular, it achieves markedly enhanced selectivity toward the desired oxidation products, highlighting its distinct advantage in modulating reaction pathways and effectively suppressing overoxidation (detailed comparisons are provided in Table S1). As depicted in Figure 2e, the selectivity for xylonic acid remained stable at 87.3% after five cycles, with a decline of less than 3%. The cumulative yield of xylonic acid reached 38.6 mmol g^−1^. There were no significant structural changes of the used catalyst through characterizations (Figures S6 and S7), suggesting the excellent stability of B‐TiO_2_ for sustained biomass conversion.

### 2.2 Photocatalyst Characterization

**FIGURE 3:**
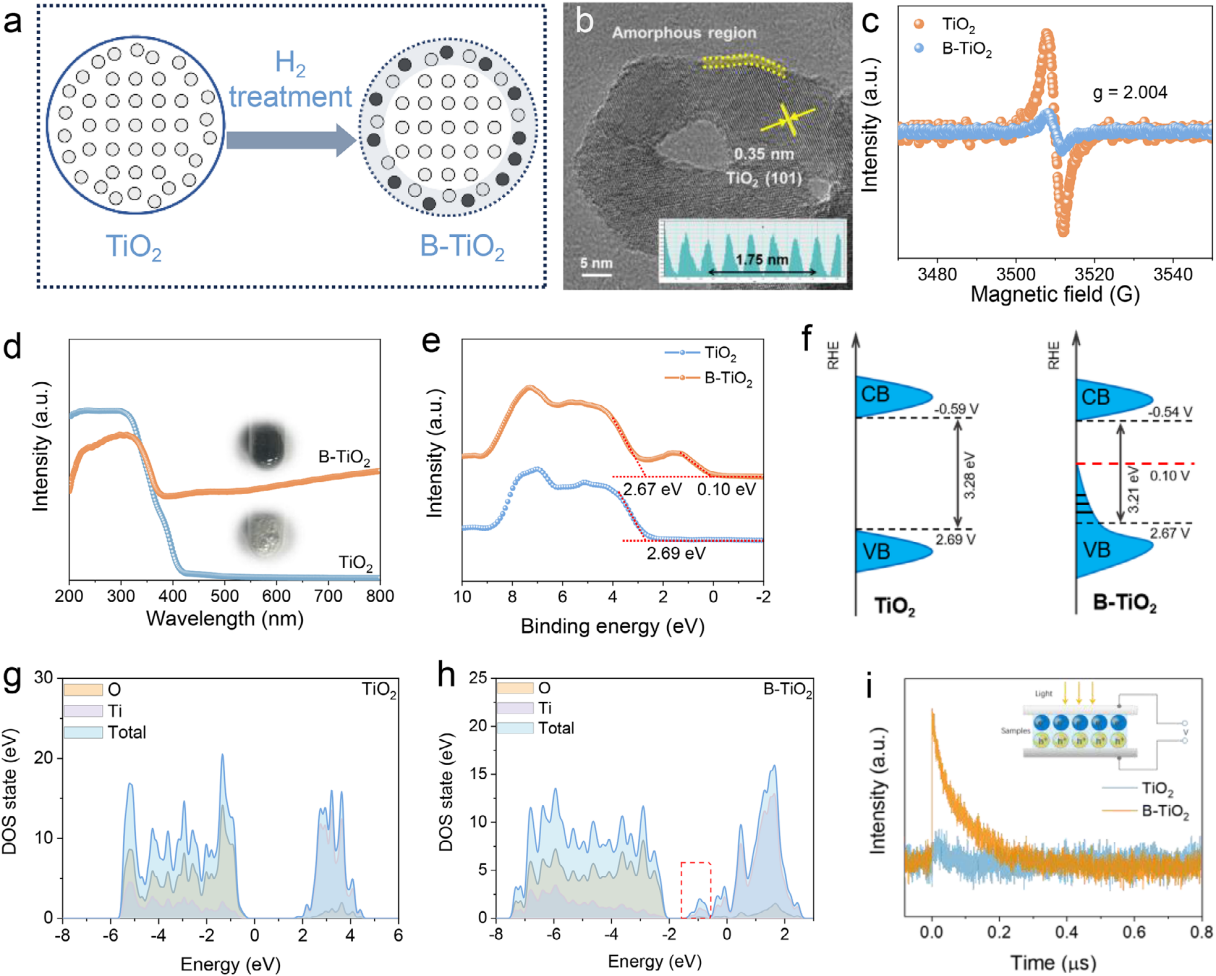
Photocatalyst characterization. (a). Schematic diagram of B‐TiO_2_ preparation (The inserted image: color comparison of TiO_2_ (white) and (black) B‐TiO_2_). (b). HRTEM image of B‐TiO_2_. (c). EPR spectra, (d). UV–vis DRS tests, (e). XPS‐VB spectra of TiO_2_ and B‐TiO_2_. (f). Energy band of TiO_2_ and B‐TiO_2_. DOS of (g). TiO_2_ and (h). TiO_2_ with O vacancies. (i). The TPV spectra of TiO_2_ and B‐TiO_2_ under 532 nm excitation.

As shown in Figure 3a, the synthesis process of B‐TiO_2_ involves a hydrogenation treatment that reduces surface Ti^4+^ to Ti^3+^ and generates oxygen vacancies [33, 34, 35]. Consequently, the color of TiO_2_ changes from white to black. B‐TiO_2_ showed particle sizes of 20–50 nm with uniformly distributed Ti and O elements in transmission electron microscopy (TEM) and element mapping images (Figures S8 and S9). High‐resolution transmission electron microscopy (HRTEM) exhibited edge amorphization and lattice fringes of 0.35 nm corresponding to the (101) plane (Figure 3b) [36, 37]. X‐ray diffraction (XRD) patterns showed characteristic peaks of anatase TiO_2_ (JCPDS 21–1272), with reduced intensities in B‐TiO_2_, suggesting diminished crystallinity induced by hydrogenation treatment (Figure S10) [26, 38]. The broadened and shifted Raman peaks further indicated the disruption of surface lattice symmetry and order (Figure S11) [39, 40]. In Figure 3c, B‐TiO_2_ showed a stronger electron paramagnetic resonance (EPR) signal than TiO_2_ due to hydrogenation‐induced generation of paramagnetic oxygen vacancies. The XPS spectrum also showed Ti^3+^ species and oxygen vacancies (Figure S12) [41, 42]. The specific surface areas of TiO_2_ and B‐TiO_2_ were 63.9 and 71.6 m^2^/g, respectively, indicating hydrogenation had little effect on surface area (Figure S13).

In Figure 3d, the UV–vis diffuse reflectance spectroscopy (UV–vis DRS) of B‐TiO_2_ exhibited markedly enhanced light absorption in the visible regions. In the Tauc plot, the band gap of B‐TiO_2_ was reduced by 0.07 eV, reaching 3.21 eV after hydrogenation treatment (Figure S14). In XPS valence band (XPS‐VB) tests, the defect energy level and VB position of B‐TiO_2_ were 0.10 and 2.67 eV, respectively (Figure 3e). For pure TiO_2_, only the VB position at 2.69 eV was observed, with no defect states detected. According to the equation *E*_g_  = * E*_VB_ − *E*_CB_, the conduction band (CB) positions of TiO_2_ and B‐TiO_2_ were located at −0.59 and −0.54 V vs. RHE, respectively (Figure 3f). Density functional theory (DFT) calculations validated the experimental results, showing that TiO_2_ with oxygen vacancies exhibited defect states in its band structure. (Figure S15). In the density of states (DOS) calculations, the introduction of oxygen vacancies affects the localization of electrons, leading to the formation of defect states near the CB and the emergence of additional electronic states, resulting in a band tail structure (Figure 3g,h).

We conducted electrochemical tests to investigate the photoelectric properties of the as‐prepared samples. The B‐TiO_2_ demonstrated a distinct surface photovoltage signal under 532 nm light excitation in transient photovoltage (TPV) spectra, indicating the visible‐light activity of the defect states (Figure 3i). In Figures S16 and S17, the enhanced transient photocurrent response and reduced charge transfer resistance of B‐TiO_2_ confirmed its improved charge transport properties. Photoluminescence (PL) measurements illustrated the enhanced photoinduced charge separation dynamics of B‐TiO_2_ (Figures S18 and S19).

### 2.3 Reaction Mechanism of B‐TiO_2_ Photocatalytic

B‐TiO_2_ with defect‐induced energy levels expands photocatalytic oxidation into the visible light region and provides a mild oxidation environment, facilitating the conversion of biomass into high‐value products and preventing the overoxidation associated with the strong oxidative capacity under UV light. Under 350 nm illumination, xylose undergoes deep oxidation with C─C bond cleavage, yielding low‐value small molecular products. In contrast, under visible light, xylose primarily undergoes aldehyde‐to‐carboxyl oxidation through the synergistic action of superoxide radicals and defect‐state holes, yielding xylonic acid with a selectivity of 90.1%. The photogenerated electrons migrate to the surface and are captured by O_2_ to generate ·O_2_^−^. Meanwhile, the holes remaining in the defect states participate in the oxidation of the aldehyde group in xylose. The holes attack the C─H bond on the aldehyde group of xylose, and the resulting xylose radical subsequently reacts with dissolved oxygen to generate xylonic acid. Simultaneously, the produced protons (H^+^) are consumed by OH^−^ to form water.

**FIGURE 4:**
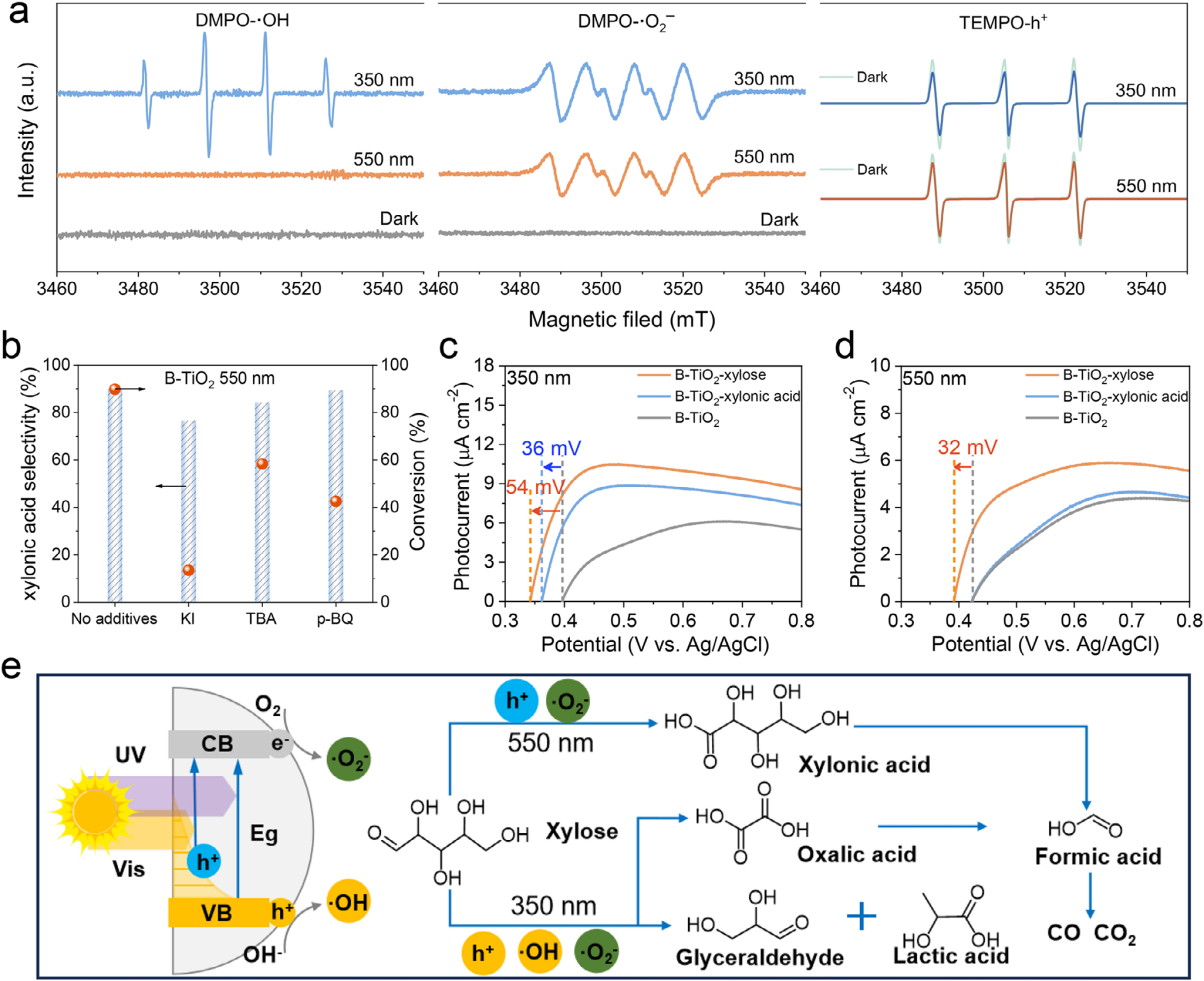
Adaptive photocatalytic mechanisms. (a). EPR spectra of DMPO‐·OH, DMPO‐·O_2_^−^ and TEMPO‐h^+^ for B‐TiO_2_ under 350 and 550 nm light illumination (DMPO: 5,5‐dimethyl‐1‐pyrroline N‐oxide, TEMPO: 2,2,6,6‐Tetramethyl‐1‐piperinedinyloxy. Light source was provided by a 300 W Xe lamp, equipped with 350 and 550 nm light filters, with a light exposure time of 5 min). (b). Radical scavenging experiments for B‐TiO_2_ under 550 nm light illumination. (KI, TBA, and p‐BQ serve as scavengers for h+, ·OH, and ·O_2_^−^, respectively. A 300 W Xe lamp with a 550 nm light filter served as the light source, and 10 mg of each scavenger was added under the same conditions as the standard photocatalytic reaction). LSV curve of B‐TiO_2_ under (c). 350 nm and (d). 550 nm light illumination. (e). Possible xylose conversion pathways.

To better understand these phenomena, we examined the active species involved in the B‐TiO_2_ reaction system. Under 350 nm illumination, signals corresponding to hydroxyl radicals (·OH), superoxide radicals (·O_2_^−^), and photogenerated holes (h^+^) were all observed in EPR spectra. Under 550 nm illumination, the photon energy (∼2.25 eV) is insufficient to induce direct valence band–conduction band excitation. Instead, electrons are preferentially excited from defect states to the CB. The oxidation potential of excited holes in, the defect energy level (∼1.71 V vs. RHE) is not sufficient to generate ·OH radicals (E(OH^−^/·OH) = 1.99 V vs. RHE), only ·O_2_^−^ and h^+^ radicals were detected in EPR spectra (Figure 4a). The highly oxidative ·OH radicals are primarily responsible for the cleavage of C─C bonds in xylose, resulting in the formation of C1 products.

To validate this mechanistic distinction, scavenger experiments were conducted under different illumination conditions. Under 350 nm irradiation, the addition of the hole scavenger potassium iodide (KI) leads to a drastic decrease (78.1%) in xylose conversion, indicating that holes are the primary species responsible for C─C bond cleavage. The introduction of the hydroxyl radical scavenger tert‐butyl alcohol (TBA) results in an 36.7% decrease in conversion, suggesting that ·OH is also a major contributor. In contrast, quenching ·O_2_^−^ reduced the conversion efficiency but exerted minimal influence on selectivity, indicating its limited involvement in C─C cleavage. (Figure S20). According to the radical scavenger experiments under 550 nm visible‐light irradiation, the addition of the hole scavenger KI resulted in a significant decrease of 76.4% in xylose conversion, indicating that defect‐state‐derived holes are the dominant active species under visible light. The introduction of the superoxide radical scavenger para‐benzoquinone (p‐BQ) led to a decrease of about 47.3% in xylose conversion, suggesting that ·O_2_^−^ also participates in the reaction, although its contribution is secondary. (Figure 4b). Control experiments were performed under an Ar atmosphere after removing dissolved oxygen to clarify the role of O_2_. Under 550 nm light irradiation, the conversion of xylose decreased markedly compared with that under O_2_‐containing conditions (Figure S21), confirming that O_2_ serves as the primary electron acceptor and that ·O_2_^−^ generated from O_2_ reduction participates in the reaction. Notably, the selectivity toward xylonic acid remained nearly unchanged, indicating that the moderately oxidative defect‐state holes are responsible for the selective oxidation of xylose to xylonic acid.

Xylonic acid, the main product under 550 nm, undergoes further oxidation under 350 nm irradiation due to the highly oxidative ·OH radicals. Comparative experiments indicate that the conversion rate of xylose acid reached 50% under 350 nm light, in contrast to only 6% under 550 nm illumination. As illustrated in Figure 4c, linear sweep voltammetry (LSV) tests further revealed that under 350 nm illumination, the addition of either xylose or xylose acid led to a negative shift in the onset potential. In comparison, only xylose induced a 32‐mV negative shift in the onset potential of B‐TiO_2_ under 550 nm irradiation (Figure 4d). Based on the results of this comprehensive study, we propose a conversion pathway for xylose under different light conditions (Figure 4e). Under UV light, highly oxidative species, especially valence‐band holes and ·OH radicals, induce deep oxidation of xylose through stepwise carbon‐chain cleavage. This process generates smaller intermediates such as lactic acid, glyceraldehyde, and oxalic acid, with oxalic acid further oxidized to formic acid, ultimately producing C1 products including formic acid, CO_2_, and CO. Under visible light, electrons reducing O_2_ to ·O_2_^−^ and defect‐state holes oxidizing xylose together form a redox cycle, enabling the selective conversion of xylose to xylonic acid.

### 2.4 Generalizability Study

**FIGURE 5:**
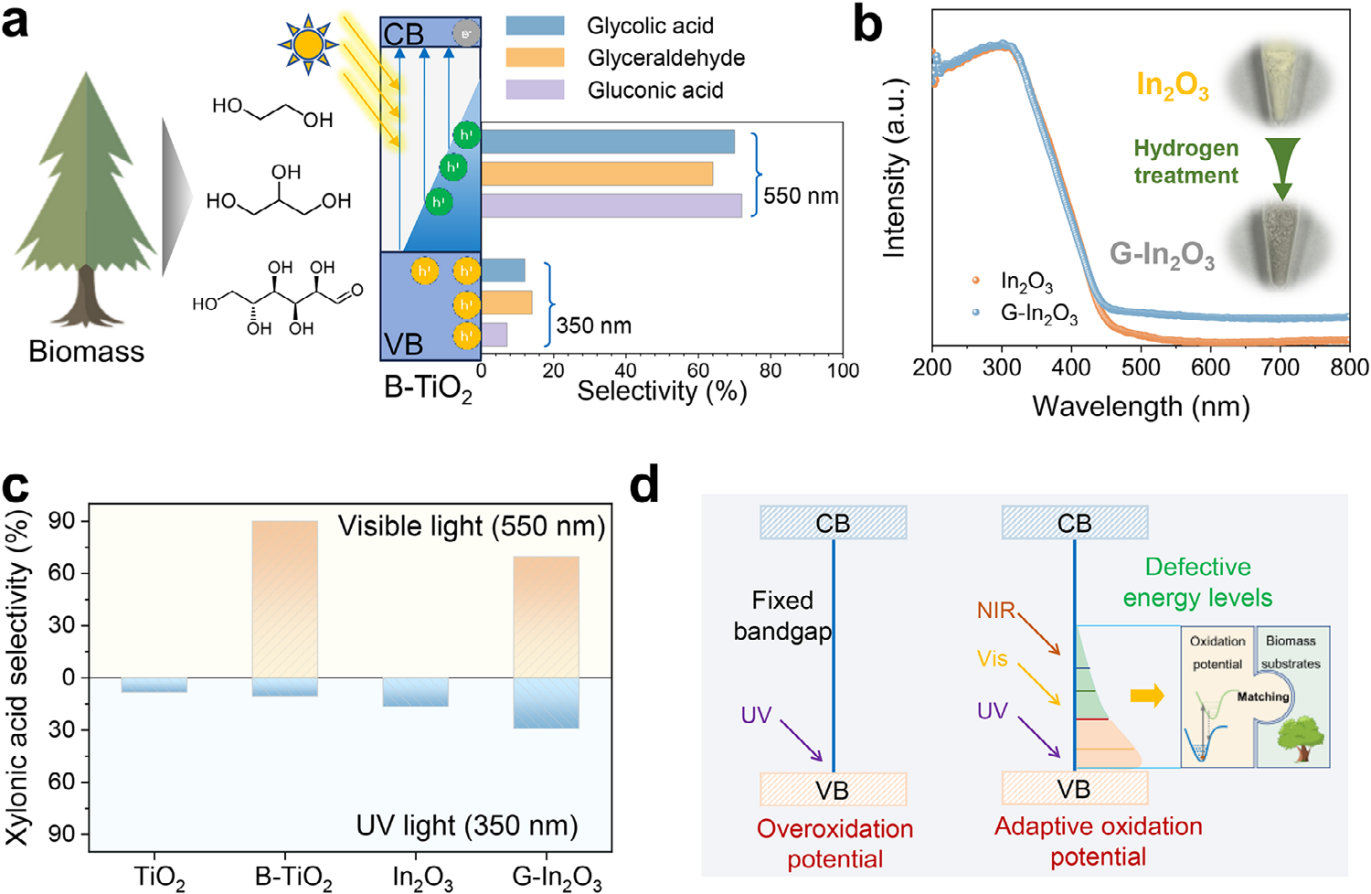
Biomass substrate conversion performance and application of adaptive strategies. (a). Photocatalytic performance of B‐TiO_2_ for different substrates. (b). UV–vis DRS tests of In_2_O_3_ and G‐In_2_O_3_. (c). Photocatalytic xylose performance of different catalysts. (d). Schematic comparison of photocatalysts with defect energy levels and those with fixed bandgaps.

To assess the generalizability of the above strategy, three representative biomass‐derived alcohols within the C2‐C6 range were selected to investigate their photocatalytic behaviors under identical conditions. As illustrated in Figure 5a, pronounced differences in product selectivity were observed under 350 and 550 nm illumination. For instance, glycolic acid, a high‐value oxidation product of ethylene glycol, exhibited a markedly enhanced selectivity of 72.0% under 550 nm light. The remaining products were predominantly C1 species, including formic acid, CO_2_, and CO under 350 nm. Analogous trends were observed for glycerol and glucose, where the selectivity toward glyceraldehyde and gluconic acid, respectively, was substantially higher under visible light excitation. Under UV illumination, glycerol was mainly oxidized to C1 products, with minor C2 products. In the case of glucose, a broader distribution of intermediate products was observed, including arabinose, erythrose, and glyceraldehyde.

This band structure engineering strategy via hydrogenation was also applied to the wide‐bandgap catalyst In_2_O_3_ (∼2.8 eV) [43, 44]. Structural and surface analyses confirmed the successful synthesis of gray In_2_O_3_ (G‐In_2_O_3_) (Figures S22–S24). EPR spectroscopy indicated the introduction of oxygen vacancies in G‐In_2_O_3_, with a markedly stronger signal compared to pristine In_2_O_3_ (Figure S25). As shown in Figure 5b, following hydrogenation treatment, the color of In_2_O_3_ changed from yellow to gray, accompanied by enhanced light absorption in the visible light region. Transient photocurrent and EIS measurements demonstrated the change of photoelectrochemical properties after hydrogenation (Figure S26). G‐In_2_O_3_ displayed a distinct transient photocurrent response under 550 nm irradiation, whereas pristine In_2_O_3_ showed no observable photocurrent under identical conditions (Figure S27). Consistently, the TPV spectra revealed a distinct surface photovoltage signal for G‐In_2_O_3_ upon visible light excitation (Figure S28). These results demonstrate that the defect states introduced by hydrogenation can be effectively excited by visible light, generating electron–hole pairs that participate in the biomass conversion process. Under illumination at different wavelengths, G‐In_2_O_3_ displayed a clear wavelength‐dependent performance in xylose conversion. Specifically, under 550 nm light, G‐In_2_O_3_ achieved the highest xylonic acid selectivity of 69.8%, significantly outperforming its performance under 350 nm illumination. In comparison, pure In_2_O_3_ exhibited only 16.5% xylonic acid selectivity under 350 nm light and showed negligible activity under longer wavelengths (Figure 5c).

The above results further demonstrate that B‐TiO_2_ with defect energy levels can self‐adjust its oxidation capacity in response to varying light conditions, thereby providing enhanced adaptability for biomass transformation. In the photocatalytic selective conversion of xylose to xylonic acid, oxygen vacancies and Ti^3+^ in B‐TiO_2_ play critical and synergistic roles. Oxygen vacancies extend the light absorption range of the catalyst into the visible region by introducing defect energy levels. They also serve as surface active sites that adsorb xylose molecules, bringing reactive species such as superoxide radicals and holes closer to the substrate and thereby facilitating the initiation of oxidation pathways. Meanwhile, Ti^3+^ acts as an electron‐enriched center that effectively captures photogenerated electrons, suppresses carrier recombination, and transfers electrons to dissolved oxygen to generate superoxide radicals, thereby enhancing the oxidation process. Beyond these static functions, the continuous distribution of defect states in B‐TiO_2_ enables dynamic, wavelength‐dependent excitation. By modulating the incident light wavelength, different defect states are selectively activated, generating distinct oxidation potentials for different biomass‐derived substrates without altering the material composition. Thus, the wavelength‐switchable defect excitation strategy introduces a light‐governed, adaptive redox capability that expands the design flexibility of selective biomass oxidation systems (Figure 5d).

## 3 Conclusion

In summary, this work demonstrates that defect‐engineered photocatalysts enable wavelength‐controlled selectivity in biomass conversion. Black TiO_2_ with defect states achieved 93.5% conversion and 90.1% selectivity for xylose‐to‐xylonic acid under 550 nm light, while UV irradiation (350 nm) led mainly to C1 by‐products. The findings emphasize the importance of tailoring defect energy levels to match the redox characteristics of target reactions, enabling mild and selective transformation pathways. The catalyst delivered a cumulative yield of 38.6 mmol g^−^^1^ with stable performance over five cycles. The strategy was further validated across different biomass‐derived substrates (ethylene glycol, glycerol, and glucose) and catalysts (gray In_2_O_3_), confirming its broad adaptability. This adaptive nature of defective photocatalysts provides a generalizable strategy to enhance photocatalytic selectivity and efficiency by rationally tuning defect states, offering new insights into catalyst design for sustainable biomass valorization.

## 4 Experimental Section

### 4.1 Photocatalyst Synthesis

Synthesis of B‐TiO_2_: The preparation method was modified based on previous reports [25]. 1 g of TiO_2_ and 1 g of NaBH_4_ were mixed thoroughly and then calcined in a tube furnace at 400°C for 6 h under a 10% H_2_/90% Ar atmosphere to obtain B‐TiO_2_.

### 4.2 Photocatalytic Xylose Oxidation

10 mg of photocatalysts were added to 10 mL 10 mm xylose solution with different KOH concentrations. The mixture was sonicated for 5 min to make a uniform dispersion of the photocatalyst. A 300 W Xe lamp equipped with different filters was used as the light source. At specified time intervals, 100 µL samples were extracted from the reactor and analyzed using HPLC (Agilent 1260 Infinity II with Shodex SUGAR SH1011 column) to detect the concentrations of xylose and products. The mobile phase in the HPLC consisted of 5 mM H_2_SO_4_, with a flow rate of 1.2 mL/min. The formulas for calculating product conversion and xylonic acid selectivity are provided below:

$$\mathrm{Conversion}\mathrm{rate}(\%)=\frac{M_{0}-M}{M_{0}}\times 100\%$$

$$\mathrm{Xylonic}\mathrm{acid}\mathrm{selectivity}(\%)=\frac{M_{product}}{M_{0}-M}\times 100\%$$

where M_0_, M, and M_Product_ represent the initial concentration of xylose, the residual concentration of xylose after the reaction, and the concentration of the product, respectively.

## Conflicts of Interest

The authors declare no conflicts of interest.

## Supporting information




**Supporting File**: advs74986‐sup‐0001‐SuppMat.docx.

## Data Availability

The data that support the findings of this study are available from the corresponding author upon reasonable request.;
