# Supplementary material for: Tuned Oxidation Potential of Continuous Defective Photocatalyst for Selective Biomass Conversion
Source: Adv Sci (Weinh). 2026 Mar 24;13(32):e23412. doi: 10.1002/advs.202523412 (PMC13252602; doi:10.1002/advs.202523412)
Supplement: Supplementary file 1 — Supporting File: advs74986‐sup‐0001‐SuppMat.docx. [file ADVS-13-e23412-s001.docx]

**Supporting Information**

**Tuned oxidation potential of continuous defective photocatalyst for selective biomass conversion**

Longfei Hong^1^†, Huiyan Zhang^1^*†, Liangdong Hu^1^, Xiang Gao^1^, Lianhua Xu^1^, Qingyu Liu^1^, Rui Xiao^1^*, Sheng Chu^1^*

^1^Key Laboratory of Energy Thermal Conversion and Control of Ministry of Education, School of Energy and Environment, Southeast University; Nanjing 210096, China.

†These authors contributed equally to this work.

* Corresponding authors E-mail: [hyzhang@seu.edu.cn](mailto:hyzhang@seu.edu.cn)

[ruixiao@seu.edu.cn](mailto:ruixiao@seu.edu.cn)

schu@seu.edu.cn

**Supplementary Text**

**Photocatalyst characterization**

The morphology of the catalyst was observed by transmission electron microscopy (TEM, Talos F200X G2 electron microscope operating at 200 kV). X-ray diffraction (XRD, Rigaku Ultima IV X-ray diffractometer (Cu Kα), scanning rate of 2° min^−1^) was used to confirm the crystal structure of the material. The X-ray photoelectron spectra (XPS) were measured on a Thermo Scientific K-Alpha with an Al Kα (h*v* = 1486.6 eV) radiation source. Raman spectra were measured on Renishaw InVia Qontor with a 532 nm laser. The specific surface area and pore-size distributions were obtained by N_2_ adsorption-desorption measurement (Micromeritics ASAP 2020 Plus 2.0). Optical absorption properties of photocatalysts were detected by UV-vis-NIR diffuse reflectance spectra (UV-vis-NIR DRS, Shimadzu UV-3600 spectrometer equipped with an integrating sphere). FLS1000 photoluminescence spectrometer was used to obtain the photoluminescence (PL) spectra. The transient photovoltage (TPV) spectra were obtained on the CEL-SPS1000.

**Electron paramagnetic resonance (EPR) spectroscopy**

EPR spectra were obtained on the Bruker EMXplus-6/1 instrument at room temperature. 10 mg of photocatalyst were dispersed in 10 mL of 50 mM KOH aqueous solution and ultrasonicated for 5 min to obtain a homogeneous suspension. For ·OH detection, 30 μL of the suspension was mixed with 30 μL of 100 mM 5,5-dimethyl-1-pyrroline-N-oxide (DMPO) and then subjected to EPR measurement. For hole detection, 100 μL of the reaction suspension was mixed with 2 μL of 100 mM 2,2,6,6-tetramethylpiperidine-1-oxide (TEMPO), followed by EPR analysis. For ·O_2_^-^ detection, 10 mg of photocatalyst was dispersed in 10 mL of 50 mM KOH methanol solution and ultrasonicated for 5 min. Then, 30 μL of the suspension was mixed with 30 μL of 100 mM DMPO and used for EPR measurement. Light source was provided by a 300 W Xe lamp, equipped with 350 nm and 550 nm light filters respectively, with a light exposure time of 5 min.

**Photoelectrochemical measurements**

The photoelectrochemical measurements were conducted on an electrochemical workstation (CHI 760E, Shanghai Chenhua Instrument Co., Ltd. China) with standard three electrodes in 0.5 M Na_2_SO_4_ electrolyte solution: Fluorine-doped tin oxide (FTO) conductive glass was employed as the working electrode, the Pt mesh and Ag/AgCl served as counter electrode and reference electrode, respectively. The working electrode was prepared as follows: 10 mg photocatalyst was dispersed in mixed solution of 1 mL ethanol and 20 μL Nafion and followed by ultrasonic treatment for 15 min. The slurry was evenly applied to fluorine-doped tin oxide (FTO) conductive glass by spin coating. Finally, the electrode was heated at 100 °C for 1 h to improve the interfacial adhesion. In photocurrent response measurements, light from a 300 W Xe lamp was irradiated from the backside of the working electrode to minimize the influence of sample thickness. The exposed area under illumination was 1 cm^2^.

**DFT computational details**

DFT calculations were performed in the framework of the density functional theory with the projector augmented plane-wave method, as implemented in the VASP. The generalized gradient approximation proposed by Perdew, Burke, and Ernzerhof screened hybrid density functional were selected for the exchange-correlation potential. The van der Waals interaction was described by the DFT-D3 approach. The cut-off energy for plane wave was set to 400 eV. The energy criterion was set to 10^-5^ eV in iterative solution of the Kohn-Sham equation. A vacuum layer of 20 Å was added perpendicular to the sheet to avoid artificial interaction between periodic images. To calculate the band structure and density of states, the Brillouin zone integration was performed using a 7 x 7 x 3 K-mesh. All the structures were relaxed until the residual forces on the atoms have declined to less than 0.02 eV/Å.


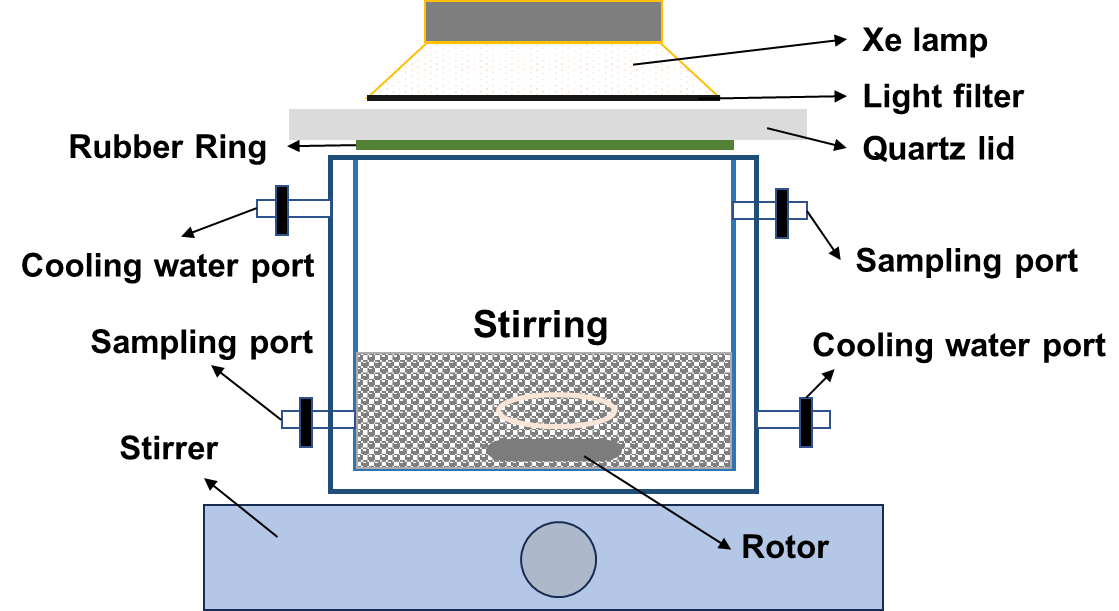


**Figure S1.** Schematic diagram of the photocatalytic reactor.

The powder photocatalyst suspension was stirred using a magnetic stirrer to ensure uniform irradiation, and optical filters were employed to adjust the incident light wavelength.

**Figure S2.** Photocatalytic performances of B-TiO_2_ under different KOH concentration.

**Figure S3.** EPR spectra of black TiO_2_ with different hydrogenation time.

**Figure S4.** Photocatalytic performance for xylose conversion under 550 nm irradiation of black TiO_2_ with different hydrogenation time. (Photocatalysts synthesis: 1 g of TiO_2_ and 1 g of NaBH_4_ were thoroughly mixed and then calcined in a tube furnace at 400 °C under a 10% H₂/90% Ar atmosphere for different time (3, 6, and 9 h) to obtain black TiO_2_ samples with varying defect concentrations. The resulting samples were denoted as B-TiO_2_ (3h), B-TiO_2_ (6h), and B-TiO_2_ (9h), respectively.)

Black TiO_2_ with varied oxygen vacancy concentrations was prepared by adjusting the hydrogenation time. With increasing vacancy concentration, xylose conversion decreased while xylonic acid selectivity increase. A moderate defect density enhances visible-light excitation and selective oxidation, whereas excessive defects promote recombination and reduce activity. The 6 h hydrogenated sample shows the optimal balance between activity and selectivity.

**Figure S5.** Photocatalytic performances of TiO_2_ under different wavelengths.

**Figure S6.** Raman spectra of fresh and used B-TiO_2_.

The main Raman peaks observed in both fresh and used B-TiO_2_ showed no detectable differences in either intensity or position.

**Figure S7.** XRD patterns of fresh and used B-TiO_2_.

The XRD patterns of B-TiO_2_ showed no observable changes after the reaction, suggesting that its crystal structure and particle size are well retained.


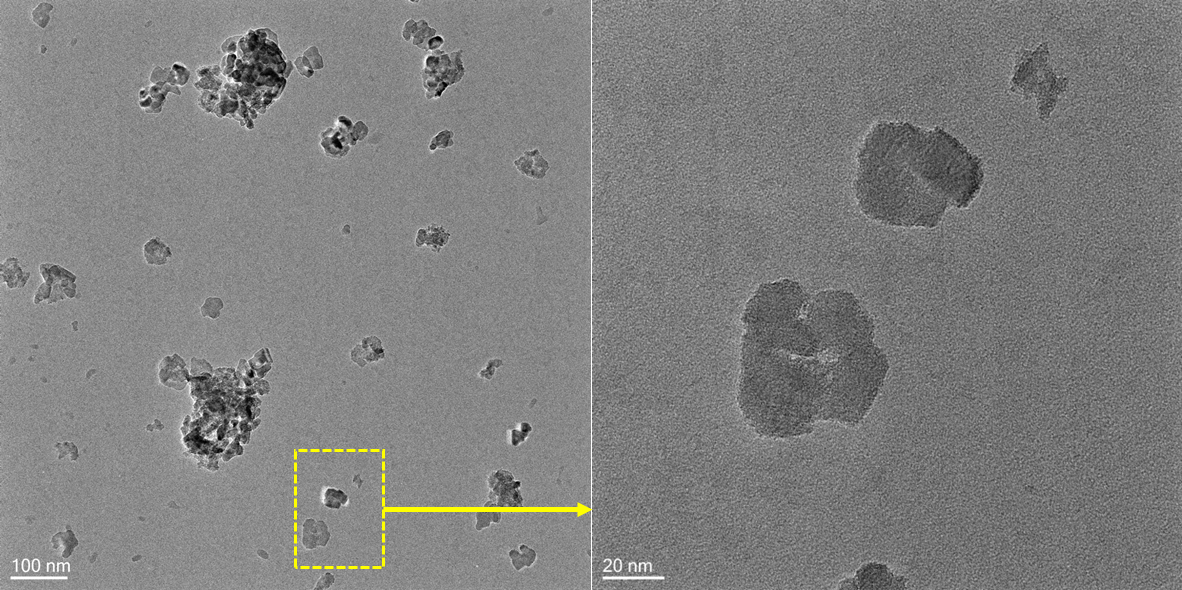


**Figure S8.** TEM images of B-TiO_2_.


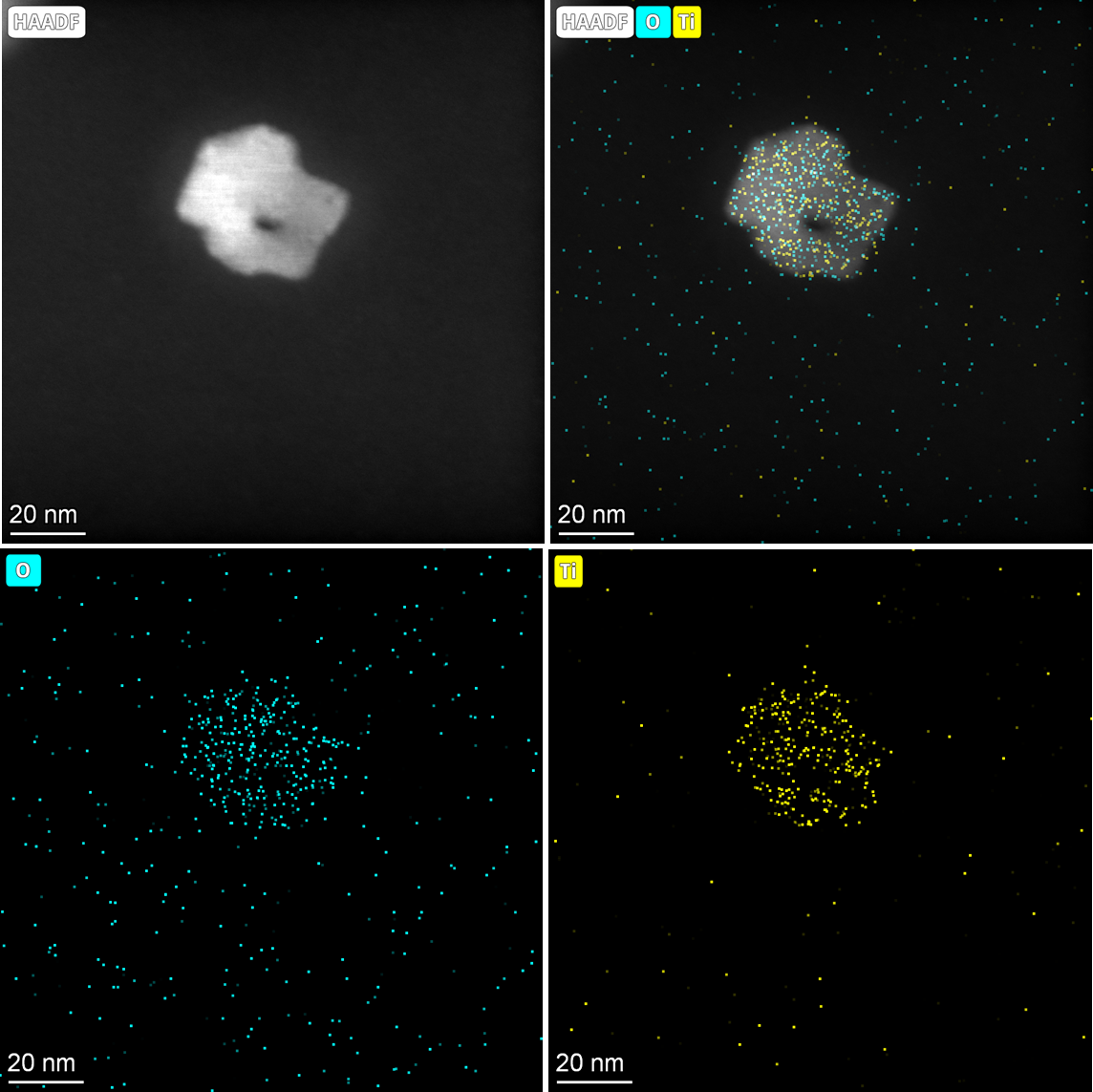


**Figure S9.** Element mapping of B-TiO_2_.

The element mapping image clearly exhibited a homogeneous distribution of Ti and O elements across the particles.

**Figure S10.** XRD patterns of TiO_2_ and B-TiO_2_.

The X-ray diffraction (XRD) patterns showed characteristic peaks at 25.4°, 37.8°, 48.1°, 54.1°, and 55.3° indexed to the anatase TiO_2_ (JCPDS 21-1272). ^1-3^

**Figure S11.** Raman spectra of TiO_2_ and B-TiO_2_.


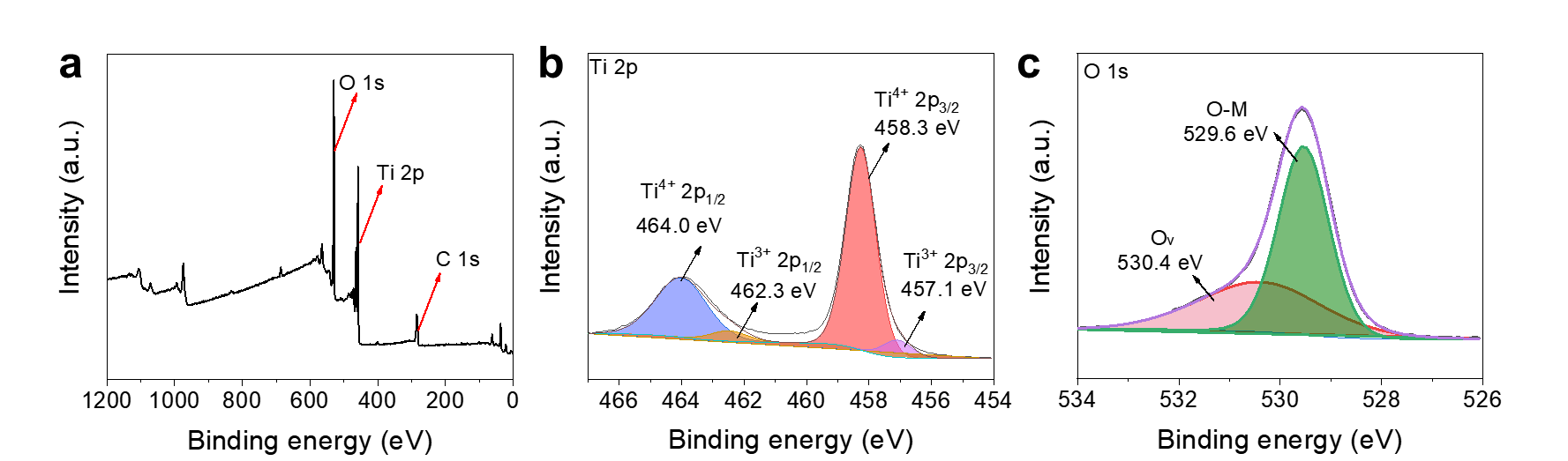


**Figure S12.** **a.** XPS survey of B-TiO_2_. **b.** Ti 2p, and **c.** O 1s high resolution XPS spectra of B-TiO_2_.

The XPS survey spectrum showed the presence of Ti and O elements (**Figure S11a**). In the Ti 2p spectrum of black TiO_2_, four peaks were observed. The peaks at 457.1 and 462.3 eV corresponded to Ti^3+^ species, while the peaks at 458.3 and 464.0 eV were attributed to Ti^4+^ (**Figure S11b**).^4,5^ As shown in **Figure S11c** , in the O1s XPS spectrum, the peaks at 529.6 and 530.4 eV corresponded to the oxygen-metal bond (O-Ti) and oxygen vacancies.


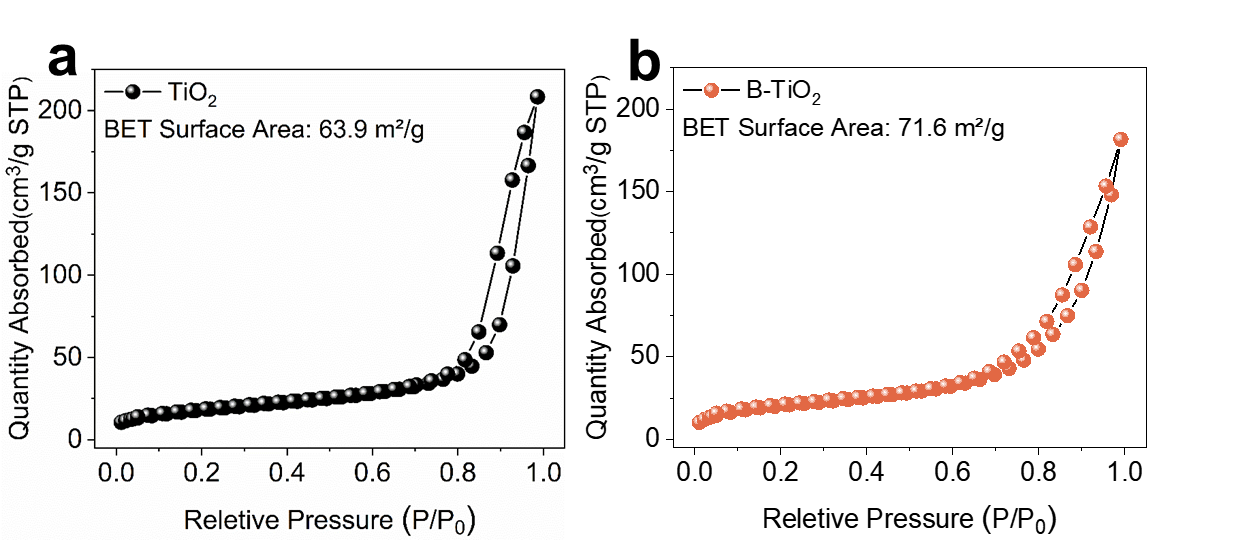


**Figure S13.** N_2_ adsorption-desorption isotherms of TiO_2_ and B-TiO_2_.

The specific surface areas of TiO_2_ and B-TiO_2_ were 63.9, and 71.6 m^2^/g, respectively, indicating that hydrogenation had a negligible impact on surface area.

**Figure S14.** Bandgap of TiO_2_ and B-TiO_2_.

The band gap of white TiO_2_ was 3.28 eV. After hydrogenation treatment, the band gap was reduced by 0.07 eV, reaching 3.21 eV.


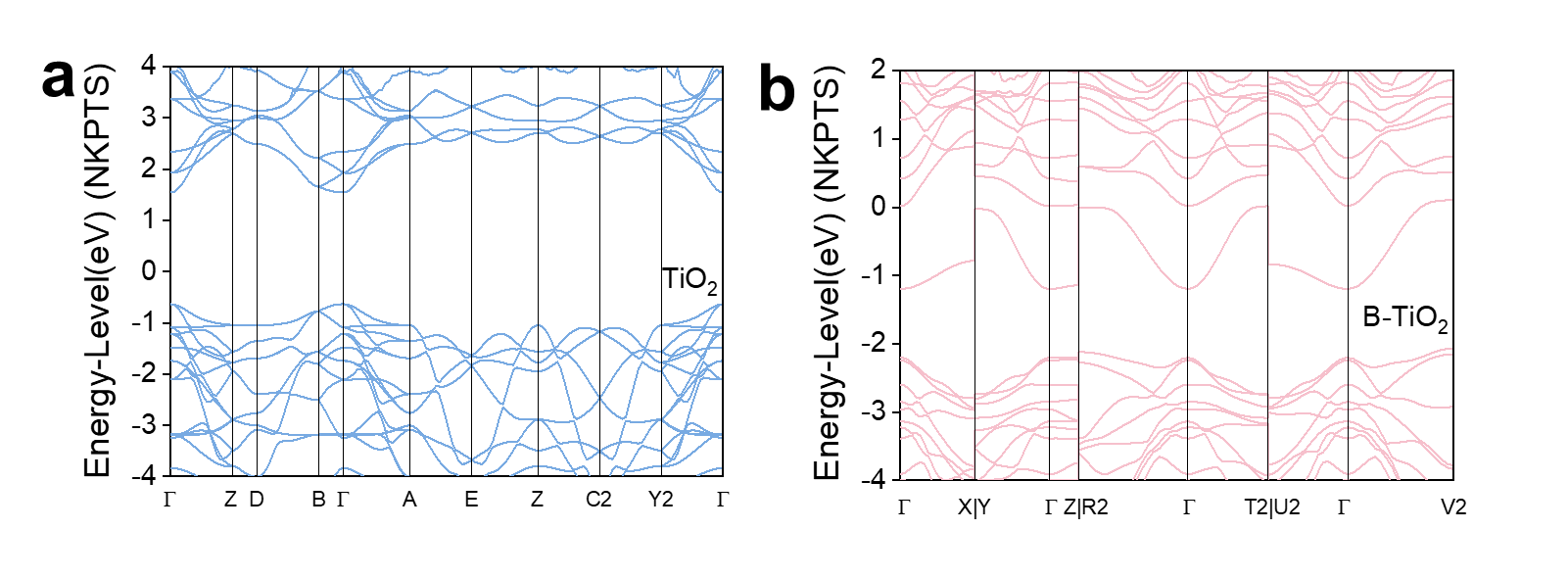


**Figure S15.** Band structures of **a.** TiO_2_ (anatase) and **b.** TiO_2_ (anatase) with O vacancies.

**Figure S16.** Photocurrent of TiO_2_ and B-TiO_2_.

**Figure S17.** EIS spectra of TiO_2_ and B-TiO_2_.

**Figure S18.** PL spectra of TiO_2_ and B-TiO_2_.

**Figure S19.** Time-resolved photoluminescence of TiO_2_ and B-TiO_2_.

**Figure S20****.** Radical scavenging experiments for B-TiO_2_ under 350 nm light illumination. (Potassium iodide (KI), tert-butyl alcohol (TBA), and para-benzoquinone (p-BQ) serve as scavengers for h**^+^**, ·OH and ·O_2_^-^, respectively. A 300 W Xe lamp equipped with 350 nm light filters was used as the light source. 10 mg of the scavenger was added to the reaction system, with other reaction conditions maintained as in the standard photocatalytic procedure.

**Figure S21.** Photocatalytic performance for xylose conversion under 550 nm light irradiation in different atmospheres.

**Figure S22.** XRD patterns of In_2_O_3_ and G-In_2_O_3_.

The peaks at 30.5°, 35.3°, 50.9°, and 60.5° corresponded to the (222), (400), (440), and (622) crystal planes, respectively (PDF# 71–2195)^6,7^.

**Figure S23.** Raman spectra of In_2_O_3_ and G-In_2_O_3_.

The Raman peaks at 129.0, 303.7, 360.8, 491.0, and 623.9 cm⁻¹ were assigned to the phonon vibration modes of the body-centered cubic (bcc) phase of In_2_O_3_. The peak at 129.0 cm⁻¹ was attributed to the fully symmetric stretching vibration of the InO_6_ octahedral units^8^.


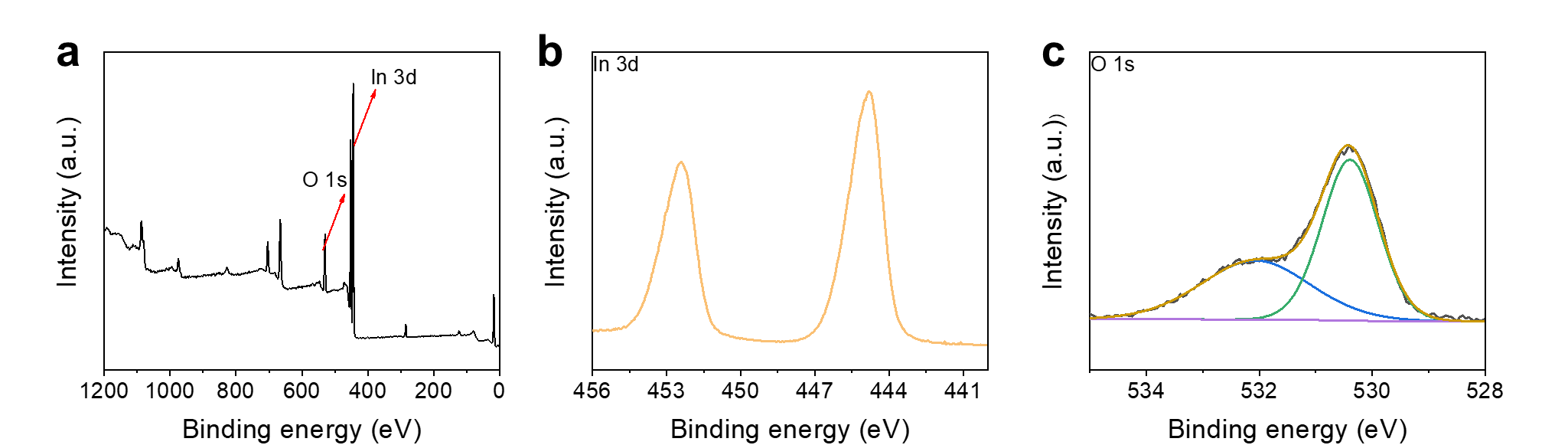


**Figure S24.** **a.** XPS survey of G-In_2_O_3_. **b.** In 3d, and **c.** O 1s high resolution XPS spectra of G-In_2_O_3_.

The In 3d spectrum displayed two characteristic peaks at 452.5 and 444.8 eV, corresponding to In 3d_3/2_ and In 3d_5/2_, respectively. In the O 1s spectrum, the peaks at 532.1 and 530.4 eV were assigned to oxygen vacancies and lattice oxygen bonded to metal ions, respectively^9^.

**Figure S25.** EPR spectra of In_2_O_3_ and G-In_2_O_3_.

**Figure S26.** EIS spectra of In_2_O_3_ and G-In_2_O_3_.

**Figure S27.** Photocurrent curves of In_2_O_3_ and G-In_2_O_3_ under 550 nm light irradiation.

**Figure S28.** The TPV spectra of In_2_O_3_ and G-In_2_O_3_ under 532 nm excitation.

**Table S1** Comparison of photocatalytic performance with the reported TiO_2_ for monosaccharide photooxidation.

| Catalyst | Poduct | Eg  (eV) | Selectivity (%) | Conversion (%) | Refs |
| --- | --- | --- | --- | --- | --- |
| TiO_2_ | Xylonic acid | 3.2 | ~58 | ~64 | ^10^ |
| TiO_2_/Ti_3_C_2_ | Xylonic acid | 3.2 | ~80 | ~82 | ^10^ |
| CuInS_2_/ZnIn_2_S_4_/g-C_3_N_4_ | Xylonic acid | 2.4 | ~60 | ~90 | ^11^ |
| AgInS_2_@CeO_2_-x | Xylonic acid | 2.78 | ~60 |  | ^12^ |
| g-C_3_N_4_-B-Cu_2_O | Lactic acid | ~2.7 | ~90 | 99.3 | ^13^ |
| Mn_x_Cd_1-x_S | Glyceric acid | 2.98 | 66 | 90 | ^14^ |
| SnO_2_/FePz(SBu)_8_ | Glucaric acid, gluconic acid and formic acid | ~3.6 | 52.2 | 34.2 | ^15^ |
| g-C_3_N_4_ | Gluconic acid | ~2.7 | ~80 | 60 | ^16^ |
| Au/C_3_N_4_ | Arabinose | ~2.7 | ~40 | ~30 | ^17^ |
| TiO_2_ | Levulinic acid | 3.2 | ~38 |  | ^18^ |
| C_3_N_4_ | Glycerol | ~2.7 | ~45 | ~95 | ^19^ |
| B-TiO_2_ | Xylonic acid | 1.1 | 90.1 | 89.9 | This work |

**References**

1 Zhang, X. *et al.* Ti^3+^ self-doped black TiO_2_ nanotubes with mesoporous nanosheet architecture as efficient solar-driven hydrogen evolution photocatalyst. *ACS Sustain. Chem. Eng.* **5**, 6894-6901 (2017).

2 Liccardo, L. *et al.* Surface defect engineering in colored TiO_2_ hollow spheres toward efficient photocatalysis. *Adv. Funct. Mater.* **33**, 2212486 (2023).

3 Chen, X., Liu, L., Yu, P. Y. & Mao, S. S. Increasing solar absorption for photocatalysis with black hydrogenated titanium dioxide nanocrystals. *Science* **331**, 746-750 (2011).

4 Xue, Z. *et al.* Understanding the injection process of hydrogen on Pt_1_-TiO_2_ surface for photocatalytic hydrogen evolution. *Appl. Catal., B* **325**, 122303 (2023).

5 Li, Y. *et al.* Deactivation and stabilization mechanism of photothermal CO_2_ hydrogenation over black TiO_2_. *ACS Sustain. Chem. Eng.* **10**, 6382-6388 (2022).

6 Deng, X. *et al.* Ultrafast electron transfer at the In_2_O_3_/Nb_2_O_5_ S-scheme interface for CO_2_ photoreduction. *Nat. Commun.* **15**, 4807 (2024).

7 Lai, K. *et al.* Photocatalytic CO_2_-to-CH_4_ conversion with ultrahigh selectivity of 95.93% on S-vacancy modulated spatial In_2_S_3_/In_2_O_3_ heterojunction. *Adv. Funct. Mater.* **34**, 2409031 (2024).

8 Wang, L. *et al.* Black indium oxide a photothermal CO_2_ hydrogenation catalyst. *Nat. Commun.* **11**, 2432 (2020).

9 Zhang, Y. *et al.* Atomically dispersed Cu on In_2_O_3_ for relay electrocatalytic conversion of nitrate and CO_2_ to urea. *ACS Nano* **18**, 25316-25324 (2024).

10 Chen, L. *et al.* Regulating TiO_2_/MXenes catalysts to promote photocatalytic performance of highly selective oxidation of d-xylose. *Green Chem.* **23**, 1382-1388 (2021).

11 Liu, K., Zhang, J., Ma, J. & Sun, R. Efficient photocatalytic conversion of xylose to co-produce xylonic acid and CO via a dual S-scheme heterojunction photocatalyst between carbon nitride and CuInS_2_ quantum dot-sensitized ZnIn_2_S_4_. *Green Chem.* **26**, 2893-2902 (2024).

12 Li, A., Ma, J., Hong, M. & Sun, R. Enhanced CeO_2_ oxygen defects decorated with AgInS_2_ quantum dots form an S-scheme heterojunction for efficient photocatalytic selective oxidation of xylose. *Appl. Catal., B* **348**, 123834 (2024).

13 Lv, Y. *et al.* Boron doping g-C_3_N_4_ supported Cu_2_O for photocatalytic reforming of xylose into lactic acid. *J. Environ. Chem. Eng.* **11**, 109981 (2023).

14 Liu, Y. *et al.* Bandgap engineering control bifunctional Mn_x_Cd_1-x_S photocatalysts selectively reforming xylose to C3 organic acids and efficient hydrogen production. *J. Colloid Interface Sci* **652**, 2066-2075 (2023).

15 Zhang, Q., Ge, Y., Yang, C., Zhang, B. & Deng, K. Enhanced photocatalytic performance for oxidation of glucose to value-added organic acids in water using iron thioporphyrazine modified SnO_2_. *Green Chem.* **21**, 5019-5029 (2019).

16 Wang, J. *et al.* In situ photo-Fenton-like tandem reaction for selective gluconic acid production from glucose photo-oxidation. *ACS Catal.* **13**, 2637-2646 (2023).

17 Wang, J. *et al.* Selective superoxide radical generation for glucose photoreforming into arabinose. *J. Energy Chem.* **74**, 324-331 (2022).

18 Abdouli, I., Dappozze, F., Eternot, M., Essayem, N. & Guillard, C. Hydrothermal process assisted by photocatalysis: Towards a novel hybrid mechanism driven glucose valorization to levulinic acid, ethylene and hydrogen. *Appl. Catal., B* **305**, 121051 (2022).

19 Wang, J. *et al.* Selective C3–C4 cleavage via glucose photoreforming under the effect of nucleophilic dimethyl sulfoxide. *ACS Catal.* **12**, 14418-14428 (2022).
